# Dermatomyositis masking late onset Pompe disease in a patient with proximal muscle weakness

**DOI:** 10.1177/22143602251385048

**Published:** 2025-10-22

**Authors:** Omar Keritam, Philipp Haas, Sigrid Klotz, Kastriot Kastrati, Martin Krenn, Matias Wagner, Timothy Hasenoehrl, Rosa Weng, Gudrun Zulehner, Gregor Kasprian, Günther Regelsberger, Hans Kiener, Ellen Gelpi, Fritz Zimprich, Hakan Cetin, Thomas Scherer, Suren Jengojan

**Affiliations:** 1 Department of Neurology, 27271 Medical University of Vienna, Vienna, Austria; 2 Comprehensive Center for Clinical Neurosciences and Mental Health, 27271 Medical University of Vienna, Vienna, Austria; 3 Department of Biomedical Imaging and Image-Guided Therapy, Division of Neuroradiology and Musculoskeletal Radiology, 27271 Medical University of Vienna, Vienna, Austria; 4 Division of Neuropathology and Neurochemistry, Department of Neurology, 27271 Medical University of Vienna, Vienna, Austria; 5 Division of Rheumatology, Department of Internal Medicine III, 27271 Medical University of Vienna, Vienna, Austria; 6 Institute of Neurogenomics, 9184 Helmholtz Centrum, Munich, Germany; 7 Institute of Human Genetics, Technical University Munich, Munich, Germany; 8 Department of Physical Medicine, Rehabilitation and Occupational Medicine, 27271 Medical University of Vienna, Vienna, Austria; 9 Department of Medicine III, Division of Endocrinology, 27271 Medical University of Vienna, Vienna, Austria

**Keywords:** late onset Pompe disease, inflammatory myopathy, dermatomyositis, case report

## Abstract

Slowly progressive proximal muscle weakness in an otherwise healthy male posed particular challenges for the treating physicians, considering the wide range of possible differentials. Here we present a case of a 52-year-old male with paraparesis, elevated creatine kinase-levels, antibodies against the Mi-2 antigen and subtle skin lesions, leading to subsequent treatment for dermatomyositis. Beyond that, exome sequencing revealed biallelic variants in the gene encoding acid alpha-glucosidase with concordant reduced enzymatic activity in fibroblasts, indicating late onset Pompe disease. Subsequently performed magnetic resonance imaging revealed a pattern of involvement typical for LOPD, but histological workup from the vastus lateralis muscle was more indicative of an immune-mediated myopathy. After treatment for dermatomyositis and Pompe disease the patient showed an improvement in skin changes and a halt in muscular weakness. In conclusion, both entities could be seen in the patient. However, early and prolonged subclinical hyper-CK-emia hinted at Pompe disease as the primary entity.

## Introduction

Differential diagnostics within the spectrum of neuromuscular disease can be very challenging. Phenotypes within this disease group are heterogeneous and clinical spectra often overlap, leading to mis- or delayed diagnosis of many cases. Weakness in the proximal portions of the extremities may be present in limb-girdle muscular dystrophy, in congenital myasthenic syndromes, idiopathic inflammatory myopathy (IMM), or late-onset Pompe disease (LOPD).^1–4^ Accompanying symptoms or co-morbidities, such as muscle pain, bulbar involvement, skin lesions, or cardiomyopathy, may be helpful in narrowing down the list of suspected disorders. Muscle biopsy and genetic testing have been very successful in solving cases with myopathies.^5,6^ In recent years it has been recommended to consider genetic testing more frequently and at a very early time point.^7,8^ We describe a case with clinical and diagnostic evidence for dermatomyositis as well as Pompe disease. Our approach to the dual diagnosis led to an individualized treatment approach.

## Case description

### Case history

In a male patient, an elevated creatine kinase (CK) level (831 U/l, upper limit of normal 190 U/l) was found incidentally at the age of 29 years, which lead to a first muscle biopsy of an upper thigh muscle (exact muscle not known). Histological analysis did not show any causative pathologies and no other test results were obtained at that time. At the age of 36 years the patient experienced prolonged muscle soreness and pain in the upper legs after a stress electrocardiogram. Slowly progressing weakness and atrophy in the proximal portions of his lower extremities started at the age of 41 years. CK levels were consistently above 1000 U/l. The patient did not suffer from any heart condition and his family history was unremarkable. The patient presented to our outpatient clinic for neuromuscular diseases at the age of 52 years. Neurological assessment revealed waddling gait, distinct bilateral atrophies in the upper thigh muscles, as well as concomitant mild paresis of the hip muscles (grade 4 on the Medical Research Council for muscle strength, MRC 4) and paresis of the knee extensors (MRC 2–3). The patient managed to walk a distance of 562 meters in the 6-min walk test (6MWT), which corresponds to 91% of the age- and gender-adjusted norm values.^9^ His gross motor function was classified as Level II (“walks with limitations”), in accordance with the Gross Motor Functional Classification System-Expanded & Revised (GMFCS-E&R).^10^ The patient's forced vital capacity (FVC) was 155% of predicted value in the upright position, and 118% in the supine position. Muscular dystrophy was initially considered as a possible cause, as the CK-emia was observed over a long period of time and muscle weakness progressed slowly, prompting genetic testing. Periungual erythema, hypermobility of the fingers, digital clubbing, Guttron's papules, and livid discoloration were confirmed shortly after that consideration, but time of onset of these symptoms was not known. Although these lesions were only subtly developed, dermatomyositis was recognized as another possible cause. Furthermore, approximately nine months after the initial presentation, the patient suffered a pulmonary embolism, and a small cerebral aneurysm was found incidentally in a routine workup.

### Diagnostic workup

The initial differential diagnosis of muscular dystrophy and subtle skin lesions prompted the treating physicians to recognize two possible pathways for the diagnostic workup, which were pursued simultaneously. On the one hand, a clear positive result for antibodies against the nuclear Mi-2α antigen (anti-Mi-2α; tested with the immunoblot *EUROLINE Autoimmune Inflammatory Myopathies 16Ag*, EUROIMMUN, Lübeck, Germany) was revealed, indicating dermatomyositis. This result was confirmed in a second test. In addition, exome sequencing, performed at the Institute of Human Genetics, Technical University of Munich, Germany, uncovered two heterozygous missense variants in the gene encoding acid alpha-glucosidase (*GAA*; NM_000152.5:c.842G > A, p.Arg281Gln, *in-silico* scores: REVEL 0,75, AlphaMissense 0,184, DANN 1, MetaLR 0,82; NM_000152.5:c.1544T > C, p.Met515Thr, *in-silico* scores: REVEL 0,68, AlphaMissense 0,769, DANN 0,89, MetaLR 0,62), both initially classified as variants of uncertain significance (VUS). No other (likely) pathogenic variant or VUS were reported after exome sequencing. The first alpha-glucosidase activity assay in leukocytes showed normal results with (10.8 nmol/mg/h, lower limit of normal 4.76 nmol/mg/h) and without (37.3 nmol/mg/h, lower limit of normal 11.72 nmol/mg/h) the inhibition of acarbose, albeit with a ratio at the lower limit of normal (ratio 0.29, lower limit of normal 0.28). Subsequently, reduced enzymatic activity was determined with tandem mass spectrometry on a dried blood spot (DBS) card (ARCHIMED Life Sciences GmbH, Vienna, Austria; 1.1 µmol/l/h; lower limit of normal 2.0 µmol/l/h), as well as with a GAA activity assay in skin fibroblasts (64 pmol/min/mg; lower limit of normal 500 pmol/min/mg). For determination of alpha-glucosidase activity in leukocytes and fibroblasts a fluorometric assay using 4-methylumbelliferyl-β-D-glucopyranoside as substrate at pH 4.0 was used.^11,12^ Hence, variants could be reclassified to “likely pathogenic” and clinical and molecular findings strongly suggested the diagnosis of Pompe disease (c.842G > A: ACMG/AMP criteria PM1, PM2_supporting, PM5, PP3, PP4_moderate; c.1544T > C: ACMG/AMP criteria PP3, PP4_moderate, PM2_supporting, PM3).^13^

**Figure 1.**
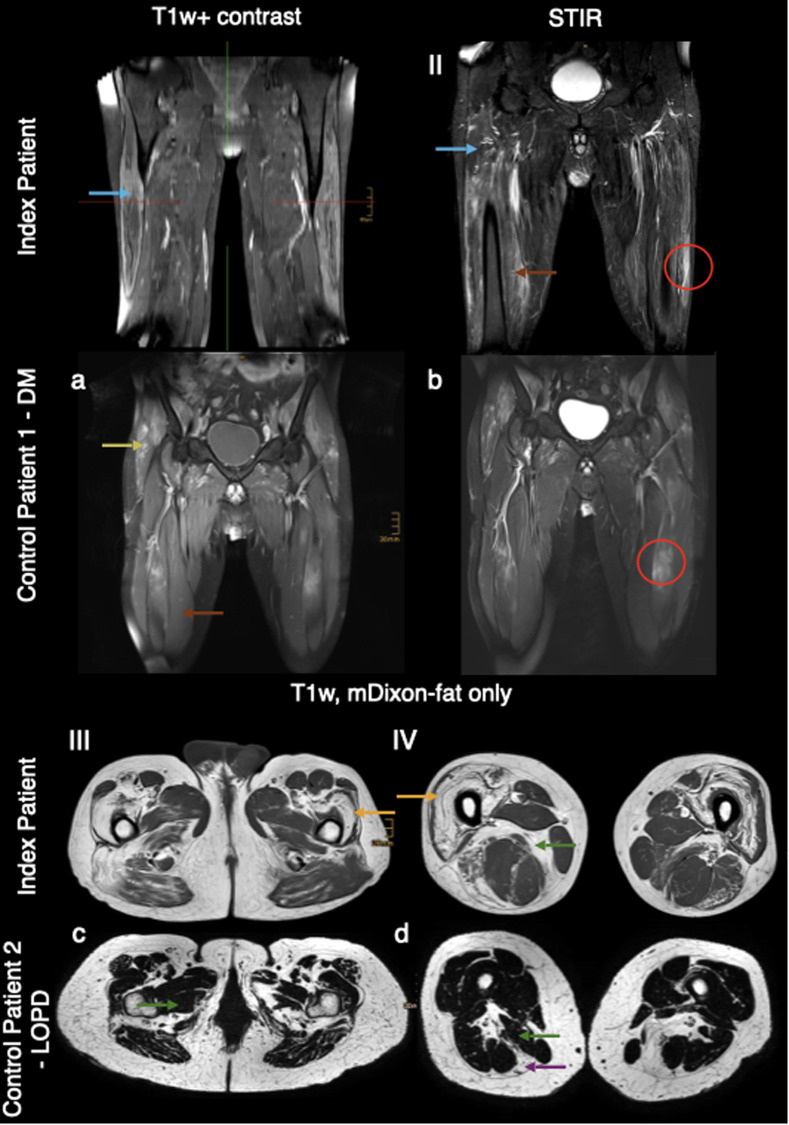
Index patient compared to patients with dermatomyositis and Pompe disease. Control patient 1 (DM) and index patient show patchy STIR-hyperintensities in the vastus lateralis muscle (blue arrow) and vastus medialis muscle (brown arrow), respectively, with corresponding contrast enhancement in the gluteus medius (yellow arrow). Red circles mark patchy edema-like regions in both patients. Control patient 2 (LOPD) and index patient show an involvement pattern typical for Pompe disease, seen as marked fat conversion of the adductor muscles (e.g., adductor magnus muscle (green arrow), semimembranosus muscle (purple arrow) and vastus lateralis (orange arrow). Note the typical involvement of the quadriceps muscle in the index patient. Compared to the index patient, control patient 2 shows less pronounced degeneration. This correlates with a less severe clinical impairment (e.g., seen in muscle strength measurement) as well as with lower creatine kinase levels. All imaging studies were performed at the Medical University of Vienna. DM = Dermatomyositis; LOPD = Late onset Pompe disease; STIR = short tau inversion recovery; T1w = T1-weighted.

**Figure 2.**
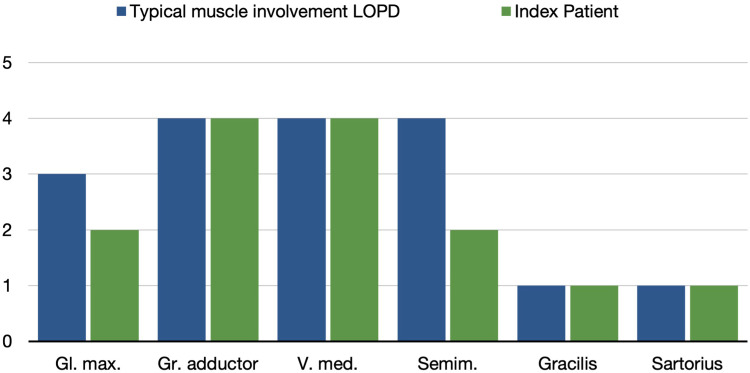
Comparison of typical muscle involvement in Pompe disease with that in the index patient. Corresponding to Carlier et al.,^25^ the involvement pattern of the lower limb muscles in Pompe patients (blue) is compared to the index patient (green). Muscle involvement was graded according to the Mercuri-score. LOPD = Late onset Pompe disease; Gl. max. = gluteus maximus muscle; Gr. adductor = great adductor muscle; V. med. = vastus medialis muscle; Semim. = semimembranosus muscle.

Magnetic resonance imaging (MRI) of the thigh muscles revealed signs suggestive of myositis as well as Pompe disease. Patchy edema-like intramuscular changes, i.e., short tau inversion recovery (STIR)-hyperintensities, were observed bilaterally in the lateral and medial vastus muscles (Figure 1). Furthermore, the MRI study showed symmetric contrast enhancement after gadolinium administration in the medial gluteus muscle. Fat conversion was found in a pattern typically seen in patients suffering from LOPD, mainly in the adductor and semimembranosus muscles (Figure 2).

**Figure 3.**
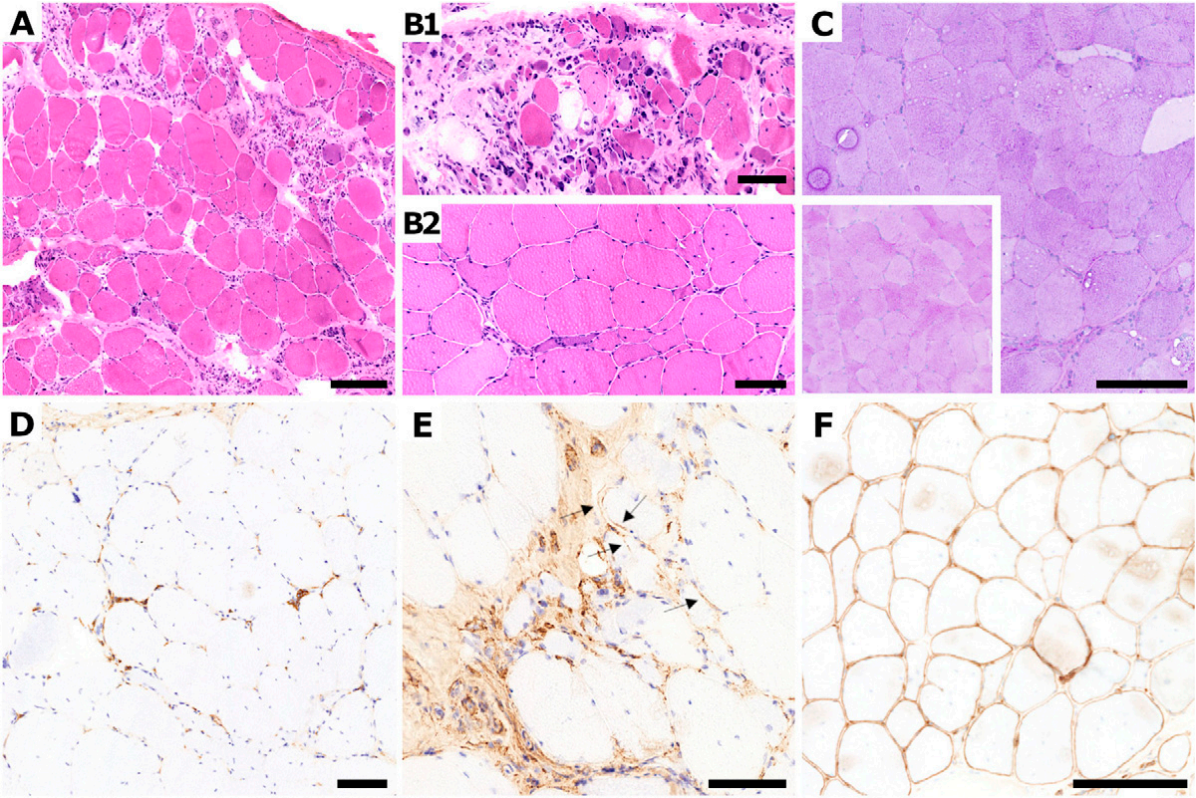
Muscle biopsy from the right vastus lateralis muscle. The histopathological analysis shows changes of a distinctive and focally accentuated myopathy (H&E, A), with some areas of perifascicular and fascicular accentuated muscle fiber atrophy (H&E, B2), with muscle fiber necrosis and myophagia (H&E, B1). These findings are associated with terminal complement deposits on the sarcolemma of individual muscle fibers and in highly atrophic areas (IHC: C5b9 [arrows], E), with only minor lymphocytic inflammatory cell infiltrates (IHC: LCA, D). The muscle shows a diffuse upregulation of N-terminal utrophin (IHC: N-terminal utrophin, F). No classic morphological changes of glycogen storage disease, such as an increase in glycogen, subsarcolemmal glycogen lakes, or vacuole formation are seen (PAS, C; normal muscle in inset). These changes, along with the presence of anti-Mi-2α antibodies, are morphologically compatible with Mi-2a-associated dermatomyositis. Scale Bars: A, C, F: 200 mm; B, D, E: 100 mm.

Initially, we were able to secure a paraffin-fixed sample from the first muscle biopsy (see case history) to repeat and re-assess the histological analysis. Hematoxylin-eosin-staining, periodic acid-Schiff reaction and immunohistochemistry did not reveal inflammatory myopathy or glycogen storage disease. Following MRI, a second biopsy was taken from the right lateral vastus muscle. The histopathological workup did not show any changes consistent with overt glycogen storage pathology but there were focal fascicular and perifascicular myopathic changes with atrophic and necrotic muscle fibers associated with terminal complement deposits along the sarcolemma of muscle fibers with only minor inflammatory cell infiltrates indicative of an inflammatory myopathy and morphologically consistent with the changes described in anti-Mi-2α-associated dermatomyositis (Figure 3).

### Therapeutic approach and follow-up

Initially, treatment for dermatomyositis was established, starting with 50 mg prednisolone, which was tapered off in the following month until a dose of 5 mg was reached. Methotrexate (MTX) was added three months after the initiation of corticosteroid treatment. In the first few months with immunosuppressive treatment, CK levels dropped significantly (474 U/l), although this effect was not retained after six months. Three months into treatment with prednisolone (two weeks before MTX treatment was initiated), however, a slight improvement in the STIR-hyperintensities was seen in a follow-up MRI. Two months into treatment with MTX, no changes in clinical status were reported and transaminases were elevated, which prompted termination of MTX. Subsequently, treatment for Pompe disease with avalglucosidase alfa (standard dose of 20 mg/kg body weight bi-weekly) was initiated, in parallel to a low-dose corticosteroid.

Long-term follow-up with MRI, which was obtained 12 months after treatment, showed no significant changes. Skin lesions improved minimally, but muscle strength, as assessed by the MRC grading system, and gross motor function did not change in a one-year follow-up. FVC values were stable in the follow-up pulmonary function test (115% upright, 107% supine). Nevertheless, the patient was able to walk 592 meters in the 6MWT at that time, which corresponded to 97% of the age- and gender-adjusted norm values and an improvement within the range of a “minimal clinically important difference”.^14^

## Discussion

Our patient presented with slowly progressing bilateral symmetric paresis and atrophies in the proximal portion of his lower extremities and with markedly and consistently elevated CK levels. Diagnostic workup hinted toward two possible and distinct entities.

Initial results revealed a strong positive anti-Mi-2α antibody signal. Anti-Mi-2α antibodies have a high positive predictive value for dermatomyositis.^15^ In addition, dermatological abnormalities that fit the profile for dermatomyositis lead the treating physicians to consider it as the most likely diagnosis at that stage, albeit the skin lesions in our patient were very mild. Assuming that the patient's syndrome could be explained by an idiopathic inflammatory myopathy (IMM), the “2017 European League Against Rheumatism/American College of Rheumatology (EULAR/ACR) classification criteria for adult and juvenile idiopathic inflammatory myopathies and their major subgroups” would suggest classifying the disorder as a “definite IMM” in the dermatomyositis subgroup.^16^

Contrary to the typical pattern for dermatomyositis, progression of muscle weakness and atrophy was very slow and developed over years, and the patient did not have any muscle pain, or accompanying heart or interstitial lung disease typical for dermatomyositis. In addition, genetic analysis revealed two heterozygous missense variants in the *GAA* gene (NM_000152.5). Unfortunately, the biallelic localization of these variants could not be confirmed genetically, as family members were not available for segregation analysis. However, both variants have a different gnomAD allele frequency and were identified in different individuals in the in-house database of the Institute of Human Genetics at the Technical University of Munich, suggesting that they may be inherited independently. According to the standards of the American College of Medical Genetics and Genomics, both variants were initially classified as variants of uncertain significance and are not listed in the Pompe disease GAA variant database (www.pompevariantdatabase.nl) but could be reclassified to “likely pathogenic” due to clinical and diagnostic evidence.^13,17^ Furthermore, c.1544T > A is listed as a pathogenic variant,^18^ supporting the reclassification of c.1544T > C as a likely pathogenic variant. Notably, the first alpha-glucosidase activity assay in leukocytes showed a result within the normal values, albeit the ratio between GAA activity with and without inhibition of acarbose was at the lower limit of normal. However, a low risk for false-negative outcomes in alpha-glucosidase activity assays in leukocytes has been previously reported.^19^ Subsequently performed assays on DBS and in cultured skin fibroblasts showed significantly reduced enzyme activity.

The initial MRI revealed bilateral symmetric intramuscular enhancement after gadolinium administration as well as STIR-hyperintensities (Figure 1). These findings are consistent with dermatomyositis,^20^ but STIR- as well as T2-hyperintensities were also previously observed in LOPD.^21–23^ Furthermore, contrast agent uptake can be found in up to 50% of non-inflammatory myopathies.^24^ Notably, our patient did not have an involvement of subcutaneous fat or fascia on MRI, which occasionally can be seen in other dermatomyositis patients.^20^ However, this does not exclude this diagnosis. On the other hand, distribution of fat conversion across muscle groups revealed a pattern typical for late-onset Pompe disease, which characteristically includes marked atrophy of the adductor and quadriceps muscles and relative sparing of the sartorius and gracilis muscles (Figure 2). In this context, the grade of muscle conversion for different muscle groups is depicted by Mercuri scores, which were similar between our patient and a cohort of patients with verified Pompe disease.^25^

Muscle biopsy from the vastus lateralis muscle was taken during the same procedure as the skin biopsy for fibroblast cultivation (see above), in which gadolinium uptake was seen in the MRI (Figure 3). Complement deposits and mild lymphocytic cell infiltrates were found in the histological workup, compatible with Mi-2α antibody-associated dermatomyositis,^26,27^ but no changes typical for glycogen storage disease, i.e., Pompe disease (Figure 3).^28^ However, in up to 30% of patients suffering from late-onset Pompe disease, histopathology may not show these classical changes.^29,30^ Histological analysis of a muscle affected by fat conversion, which did not appear enhanced upon gadolinium infusion in MRI, may have revealed changes more compatible with Pompe disease. Subsequently, the patient did not show extension of fat conversion after treatment with avalglucosidase alfa in the follow-up examination, which is in accordance with other LOPD patients treated at our clinic. However, remission from dermatomyositis could not be ruled out either.

In conclusion, the diagnostic workup was indicative of dermatomyositis as well as LOPD. The initial drop in CK levels after treatment initiation was not retained and a follow-up MRI (not shown) two months after treatment initiation showed only minor improvement. However, skin lesions minimal improvement of skin lesions were observable under anti-inflammatory treatment and muscle weakness did not worsen with corticosteroid and enzyme replacement therapy over a sum treatment time of 12 months. Additionally, results from the 6MWT showed minimal improvement in a one-year follow-up.

As some clinical and laboratory features of Pompe disease and inflammatory myopathy overlap, some reports on patients with Pompe disease initially misdiagnosed with inflammatory myositis have been previously published.^31,32^ On the other hand, we only found one report on concurrent LOPD and inflammatory myopathy,^33^ in whom inclusion body myositis (IBM) was suggested by characteristic muscle pathology findings and anti-cN1A while LOPD was genetically conformed. However, various cases with “double-trouble” diagnoses within the neuromuscular disease spectrum, based on unusual clinical or diagnostic findings, have been described in the literature. In one report, reducing body myopathy was diagnosed in a patient with antibodies against 3-hydroxy-3-methylglutaryl-coenzyme A reductase after little response to immunotherapy.^34^ In a case series on patients with inherited myopathies suffering from a second muscular disorder, dual diagnoses of CAV3-myopathy and (IBM) or mitochondrial myopathy and idiopathic myositis, for example, were based on unusual patient histories, as well as genetic and histopathological testing.^35^ Myasthenia gravis (MG) has been reported to co-exist with facioscapulohumeral muscular dystrophy or myotonic dystrophy.^36,37^ These reports highlight the importance of reassessing unusual clinical presentations or diagnostic findings, even if they suggest an extremely improbable result. Notably, the prevalence of Pompe disease is around 1:18.000,^38,39^ and that of dermatomyositis is around 1:10.000.^40^ Thus, the probability to find both diseases in the same patient may be approximately 1:180.000.000, rendering the scarcity of similar cases in the literature an untenable argument for or against a mechanistic link.

Relatively early subclinical hyper-CK-emia suggests a prolonged process, hinting at LOPD as the first causative entity. Muscle degeneration over the years could have resulted in release and presentation of the nuclear antigen Mi-2α, leading to antibody production as an “epiphenomenon” and a higher probability to develop dermatomyositis. Importantly, neuromuscular symptoms caused by dermatomyositis might have been overlapped by the already existing LOPD, which could explain the absence of a significant improvement in the functional status after appropriate anti-inflammatory treatment.

In summary, we present a case with concurrent LOPD and dermatomyositis, underscoring the importance of a rigorous characterization of disease phenotypes and diagnostic workup in patients with neuromuscular disorders. In cases with inflammatory myopathies, sub-optimal treatment responses should lead to a re-consideration of the working hypothesis, possibly resulting in genetic testing. In general, irregular clinical and diagnostic findings, an unusual patient history, or an unsatisfactory treatment outcome, should be explored for an alternative explanation.
